# Effect of β-resin of binderless coke on mechanical properties of high-density carbon blocks at high molding pressure

**DOI:** 10.1038/s41598-022-16648-8

**Published:** 2022-07-19

**Authors:** Seungjoo Park, Seon Ho Lee, Song Mi Lee, Doo-Hwan Jung

**Affiliations:** 1 Fuel Cell Laboratory, Korea Institute of Energy Research (KIER), Daejeon, 34129 Republic of Korea; 2 Energy Engineering, University of Science and Technology (UST), Daejeon, 34113 Republic of Korea

**Keywords:** Structural materials, Mechanical engineering

## Abstract

High-density carbon blocks have excellent mechanical, thermal, and electrical properties. In particular, these blocks are applied in various fields while maintaining excellent physical properties even in harsh environments. In this study, binderless coke manufactured under certain conditions was used to form green bodies (GBs) under various pressure conditions of 50 to 250 MPa, and the bodies were carbonized to form a high-density carbon block (CB). Then, the effect of the β-resin and oxygen functional groups of binderless coke on the mechanical properties of the high-density carbon block according to molding pressure was considered. When molding at a pressure of under 200 MPa, the ratio of O and C (O/C) has a greater effect, and the larger the O/C, the higher the mechanical properties. On the other hand, when molding at a high pressure of 250 MPa, the β-resin content has a greater effect and steadily increases when the β-resin content is low and when the mechanical properties are sufficiently reduced. In particular, in the case of CB-N7A3–250, which has the highest β-resin content of 3.7 wt%, the density was 1.79 g/cm3, the flexural strength was 106 MPa, and the shore hardness was 99 HSD.

## Introduction

High-density carbon blocks are much lighter than metals and have excellent mechanical, thermal, and electrical properties. In particular, they maintain excellent physical properties even in harsh environments, such as ultrahigh temperature, high pressure, and chemical composition. As a result, high-density carbon blocks are used in automobiles, aircraft, rockets, etc., used to improve fuel efficiency and are also used in various heat-dissipating materials, heat insulating materials, electromagnetic interference (EMI) shielding materials, etc. based on their excellent electrical and thermal characteristics^1–6^.

Raw materials for producing high-density carbon blocks can be divided into primary and binary materials according to the number. First, mesocarbon microbeads (MCMBs) are a typical example of primary materials, which are substances that have self-sinterability and can be molded without the need for additional binder materials^7–9^. This is because it voluntarily has binder materials called β-resin. β-resin can be defined from the difference in solubility depending on the type of solvent and generally refers to the difference in solubility between quinoline and toluene. In other words, a substance that is soluble in quinoline and insoluble in toluene is called β-resin^10^. These substances have a fluid phase and can fill the empty space between the solid phases and attach them tightly. In addition, volume shrinkage is caused during sintering, and the density can be increased^11–13^. On the other hand, since binary materials do not contain β-resin, binder materials are absolutely necessary during molding, and representative materials include highly crystalline carbon materials such as needle coke and graphite^14,15^. These materials are carbonized and then impregnated to improve their mechanical properties^3,8^. In addition, carbon nanotubes (CNTs), carbon fibers, carbon black, etc. are added to improve specific physical properties, such as electric conductivity and thermal conductivity^16–20^.

Using these raw materials, a green body is produced by cold pressing or hot pressing. Then, a high-density carbon block is manufactured through a carbonization process while being heat-treated from 800 to 1500 °C and a graphitization process while being heat-treated at over 2000 °C^21^.

One of the biggest problems in the manufacturing process of carbon blocks is the swelling phenomenon^22^. Swelling occurs by rapidly releasing volatile matter in the green body, and pores are formed^23^. Due to the swelling phenomenon, porosity increases and mechanical properties decrease. Therefore, much research has been done to prevent this problem. Mochida et al. reported that the raw material was oxidatively stabilized before molding, the test piece was not deformed even at high temperatures, volatile matter was removed so that swelling was suppressed, and then molding and heat treatment were performed^4,25–27^. In addition, Ragan et al. oxidized needle-coke to give a hydroxyl group, a carbonyl group, a carboxyl group, etc. that can contribute to the bonding strength, mixed with a coal tar binder pitch and subjected to molding and heat treatment. Then, it was reported that the amount of oxygen functional groups and the amount of escaped oxygen were compared according to the degree of oxidation of needle coke, and high mechanical properties appeared when the needle coke was molded with the most oxygen functional groups^28^. In addition, volatile components of low molecule substances can be removed by vacuum heat treatment^29^.

In previous study, in the process of manufacturing binerless coke to suppress the swelling phenomenon, a gas mixed with nitrogen and air was blown as a carrier gas to increase the molecular weight through cross-linking of low molecular weight substances. An increase in molecular weight was indirectly shown through changes in the solubility of toluene and quinoline and the fixed carbon content through proximate analysis. In addition, using XPS, it was confirmed that oxygen was taken up and the O function groups were developed, and it was confirmed that the swelling phenomenon was suppressed as the flow rate ratio of air increased by SEM. Then, the density, flexural strength and shore hardness are measured, and it is reported that the mechanical properties increase^30^.

In this study, binderless coke, which is one of the priority materials, was produced by heat treatment at 470 °C while flowing a mixed gas of air and nitrogen from a coal tar pitch. Then, using this material, a high-density carbon block was produced by cold pressing at 50 to 250 MPa. After that, the β-resin content contained in the binderless coke was measured, and the atomic % of O and C of the binderless coke was measured using X-ray photoelectric spectroscopy (XPS) to derive the O/C value. Then, the changes in the contents of fixed carbon and volatiles were investigated via proximate analysis (PA). Finally, the effect of β-resin and oxygen functional groups of binderless coke on the mechanical properties of high-density carbon blocks was analyzed according to the molding pressure.

## Materials and methods

### Binderless coke

The physical characteristics of the binderless coke used in this study are shown in Table 1^30^. As shown in the table, as the flow rate of air increases, the content of volatiles decreases and the content of fixed carbon gradually increases, showing the typical characteristics of coke produced through air blowing^31^. In addition, β-resin peaked at 3.7 wt% in N7A3 and then decreased from 1.2 wt% to 0.4 wt% in the order of N5A5, N3A7, and N0A10.

**Table 1:** Properties of binderless cokes manufactured according to the flow ratio of nitrogen and air.

Sample name	Proximate analysis (wt%)					β-resin (wt%)	
	M^a^	V.M^b^	Ash	F.C^c^	TI^d^	QI^e^	β-resin
N10A0f.^)^	1.30	8.61	0.65	89.44	97.2	94.5	2.7
N7A3	1.26	7.35	0.43	90.96	99.1	95.4	3.7
N5A5	1.13	7.34	0.40	91.13	99.4	98.2	1.2
N3A7	1.55	6.69	0.37	91.39	99.5	99.0	0.5
N0A10	1.57	6.58	0.39	91.46	99.7	99.3	0.4

^a^Moistrue.

^b^Volatile matter.

^c^Fixed carbon.

^d^Toluene insoluble.

^e^Quinoline insoluble.

^f^N_2_ 100 cc/min/Air 0 cc/min.

### Preparation of green body and carbonization

To analyze the change in the mechanical properties of the green body due to the molding pressure, the green body is manufactured from 50 to 250 MPa at room temperature. At this time, a mold of 35 × 35 × 40 mm size was used. The prepared green body was carbonized at 1200℃ for 1 h in a nitrogen atmosphere at a heating rate of 10 °C/min.

### Analysis

#### Proximate analysis

Proximate analysis was measured using thermogravimetric analysis (TGA, STA409PC, Netzsch Corp, Germany), and the analysis was performed by quoting the international standard KS E ISO 1171^30^. The content of β-resin was checked by ASTM D2318–15^32^ and ASTM D4072-98^33^.

#### O/C ratio analysis

Hydroxy group (–OH), carbonyl group (–C=O), and carboxyl group (–COOH), etc. are generated when air is added to the coal tar pitch for reaction^34,35^. Using X-ray photoelectron spectroscopy (XPS, K-alpha + , Thermo Scientific, USA), the atomic % of O and C from the hydroxy group (–OH), carbonyl group (–C=O), and carboxy group (–COOH) imparted to the surface was measured, and the O/C ratio was determined^30^.

#### Mechanical properties

Flexural strength was measured using a Universal Testing Machine (UTM, WL2100, WITHILAB ltd, Korea) with reference to ASTM D790–17^36^. Shore hardness was measured using a shore hardness tester (SH, Type-D, Kobunshi Keiki, Japan) with ASTM D2240^37^.

### Porosity analysis

Apparent density and true density were measured to measure the porosity. Apparent density was measured using the Archimedes method, and true density was measured using a pycnometer (BEL pycno, MicrotracBEL). And porosity is calculated by equation^1^ where ρ is density.

1 $$\emptyset \left( {{\text{porosity}},{\text{ \% }}} \right) = \frac{{\rho_{true} - \rho_{apparent} }}{{\rho_{true} }} \times 100$$

## Results and discussion

### Effect of molding pressure on green density

**Figure 1:**
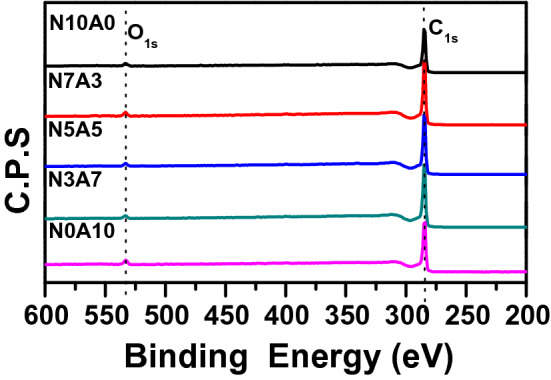
XPS graph depending on content of air flow.

**Figure 2:**
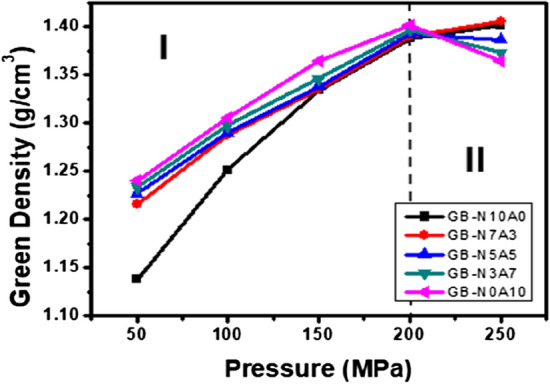
Green density changes due to molding pressure at a constant air flow rate.

**Figure 3:**
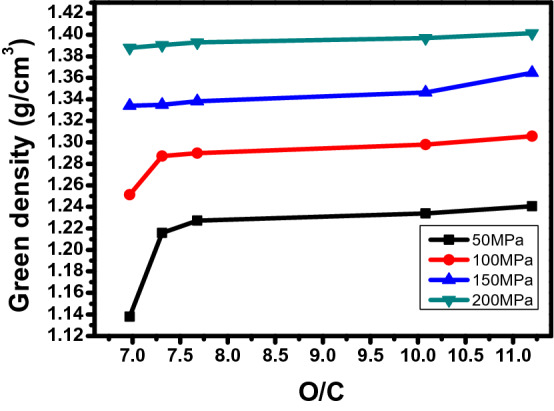
Green density change due to O/C change at constant molding pressure.

Figures 1 and 2 shows the change in green density due to molding pressure. The green body is density of green body before carbonization. The green density due to pressure is clearly displayed in the two regions. In region I, 50–200 MPa, the green density gradually increases to 1.38 g/cm^3^ as the pressure increases, but at 250 MPa, the green density decreases or the rate of increase is very weak. Table 2 and Fig. 1 show the XPS results of analyzing the oxygen functional groups of the binderless cokes used in this research^29^. As shown in Table 2 and Fig. 1, the oxygen content in the binderless cokes increases as the proportion of injected air increases, and the O1s/C1s value increases from 6.97 to 11.20, with an increase of 38%. Figure 3 shows the change in the green density depending on the O/C at a constant molding pressure. When the molding pressure is under 100 MPa and the O/C is under 7.3, the green density is very low, but when the O/C is over 7.5, the green density value is generally slightly increased. However, when the manufacturing pressure is 250 MPa, the green density decreases as the O/C ratio increases. This means that the green density is very closely related to the molding pressure and O/C and β-resin contents^13,30,38–40^.

**Table 2:** Atomic % of C1s and O1s from XPS.

Sample name	C1s (%)				O1s (%)			O/C
	C–C (C1s)	O–C=O (C1s)	C–O (C1s)	C1s (total)	O–C=O (O1s)	C–O (O1s)	O1s (total)	
N10A0	64.66	2.93	25.88	93.47	0.64	5.89	6.53	6.97
N7A3	58.93	2.87	31.39	93.19	1.52	5.29	6.81	7.31
N5A5	44.24	3.87	44.76	92.87	0.23	6.90	7.13	7.68
N3A7	42.62	4.10	42.12	90.84	1.92	7.24	9.16	10.08
N0A10	45.47	4.33	40.14	89.94	2.43	7.64	10.07	11.20

### Effect of β-resin content on green density at high molding pressure

**Figure 4:**
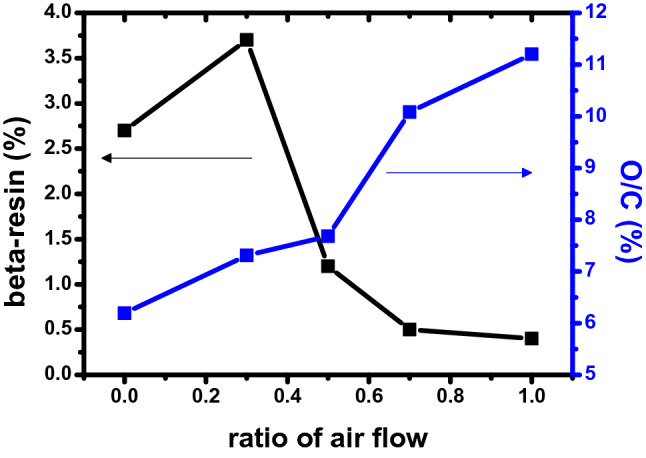
Changes in β-resin and O/C of binderless coke manufactured based on the air flow ratio (N_2_/air).

Figure 4 graphs the changes in β-resin and O/C of the binderless coke produced according to the air flow rate analyzed in Tables 1 and 2. The β-resin of the binderless coke increases to 3.7 wt% until the air ratio reaches 0.3 and then decreases to 0.4 wt%. On the other hand, the O/C steadily increases as the proportion of air increases, and it is often shown using graphs that the relationship between β-resin and O/C is trade-off. This is because as the flow rate of air increases, oxygen excessively crosslinks the small molecule substance, and the content of the β-resin having an intermediate molecular weight decreases so that it is present at a high molecular weight. These properties have a great influence on the physical properties of high-density carbon blocks and show the highest mechanical strength values when the air ratio is 1.0 at a molding pressure of 150 MPa, which has been discussed in previous studies^30^.

**Figure 5:**
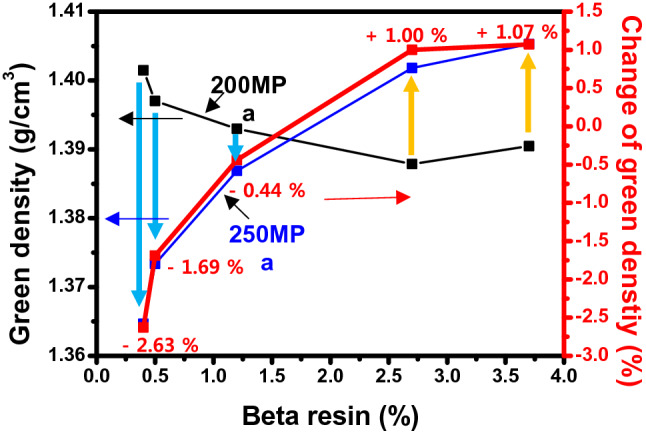
Changes in green density depending on the β-resin content.

Figure 5 shows the change in green density in region II in Fig. 2 according to the β-resin content. N10A0 and N7A3, which have β-resin contents of 2.7 and 3.7 wt%, respectively, have increased green density. On the other hand, in the case of N5A5, N3A7 and N0A10 having a low β-resin content of 1.2 wt% or less, the green density decreases when molded at high pressure. In particular, GB-N0A10 decreased by 2.63%. This is because the β-resin existing in the binderless coke leaked to the outside and did not act as a binder when molded at 250 MPa. However, in the case of GB-N10A0–250 and GB-N7A3–250, the densities increase by 1.0% and 1.2%, respectively, despite molding at 250 MPa. This is because, as shown in Fig. 4, there is sufficient β-resin in the binderless coke, so it plays a sufficient role as a binder. From this, it can be judged that the β-resin content, not the O/C, has a great influence on the mechanical characteristics in the high-pressure region of over 200 MPa.

### Carbonization characteristics according to O/C

**Figure 6:**
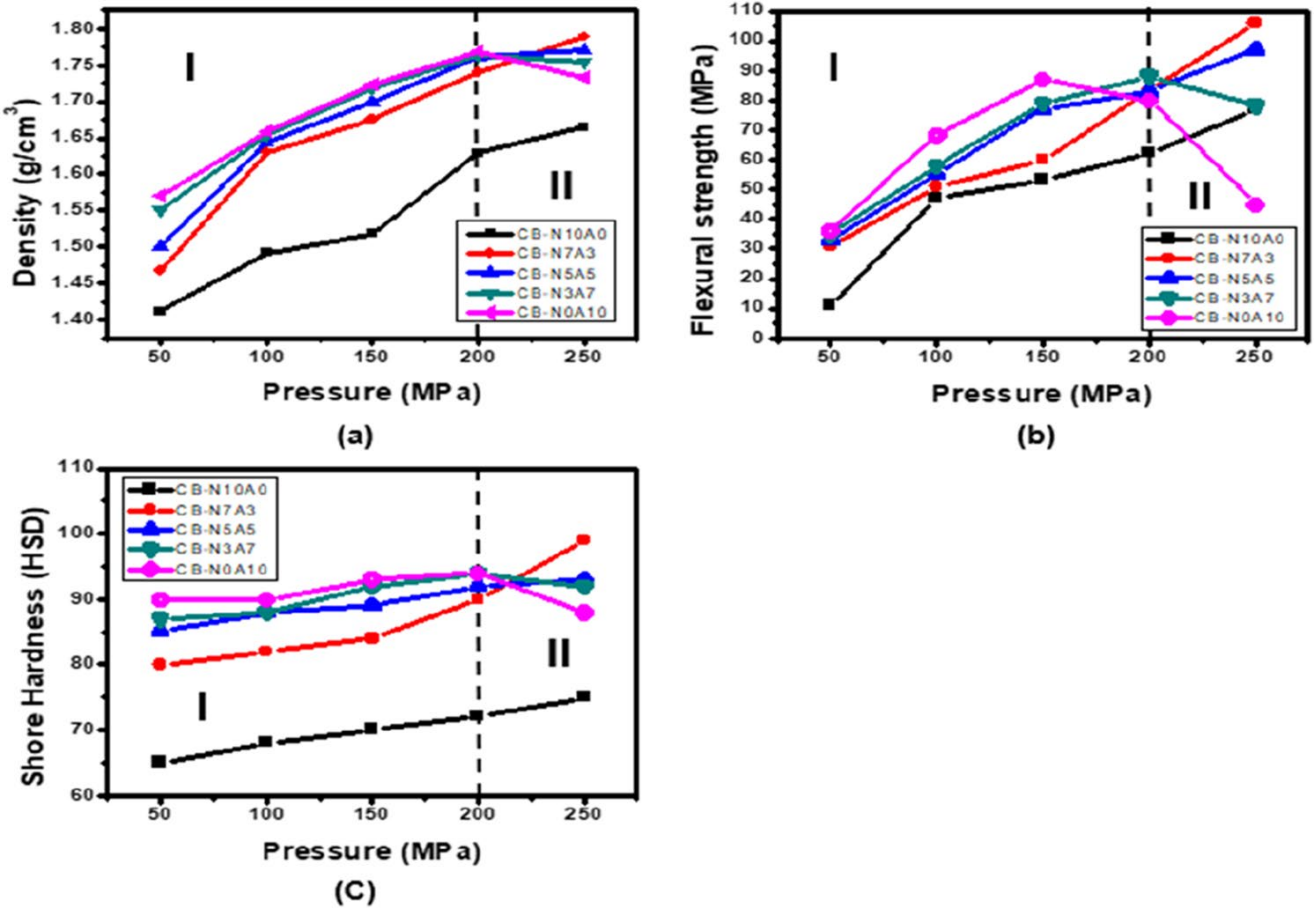
Changes in (**a**) density, (**b**) flexural strength, and (**c**) shore hardness due to the molding pressure of the high-density carbon block after carbonization.

**Figure 7:**
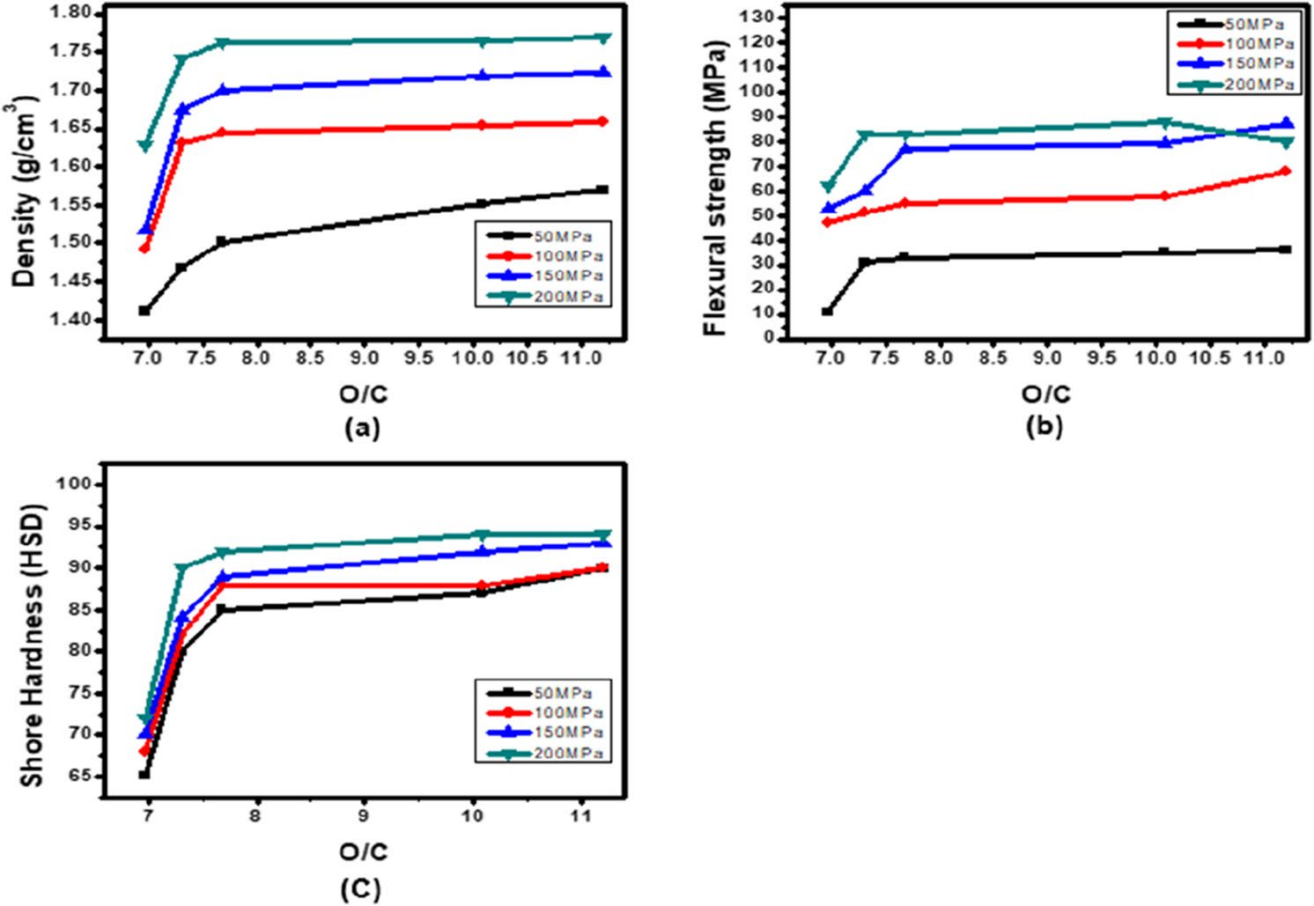
Changes in (**a**) density, (**b**) flexural strength, and (**c**) shore hardness due to O/C of the high-density carbon block after carbonization.

Figure 6 shows changes in density, flexural strength and shore hardness after carbonization. Similar to Fig. 1, Fig. 6 can be divided into two areas, one in which each mechanical property increases and the other in which it does not, based on 200 MPa. Figure 7 shows the changes in the mechanical properties of the carbon block after carbonization of region I in Fig. 6 by O/C. In the low molding pressure region I of 200 MPa or less, the mechanical properties after carbonization increase as the molding pressure increases. This shows almost the same tendency as the tendency of green density. From this tendency, it can be seen that when the green density is high, high mechanical properties after carbonization can be ensured^11,41^. At a pressure of 200 MPa or less, the mechanical properties increase after carbonization as the oxygen content increases. These trends are consistent with previous papers^30^.

### Effect of β-resin on mechanical properties at high molding pressure

**Figure 8:**
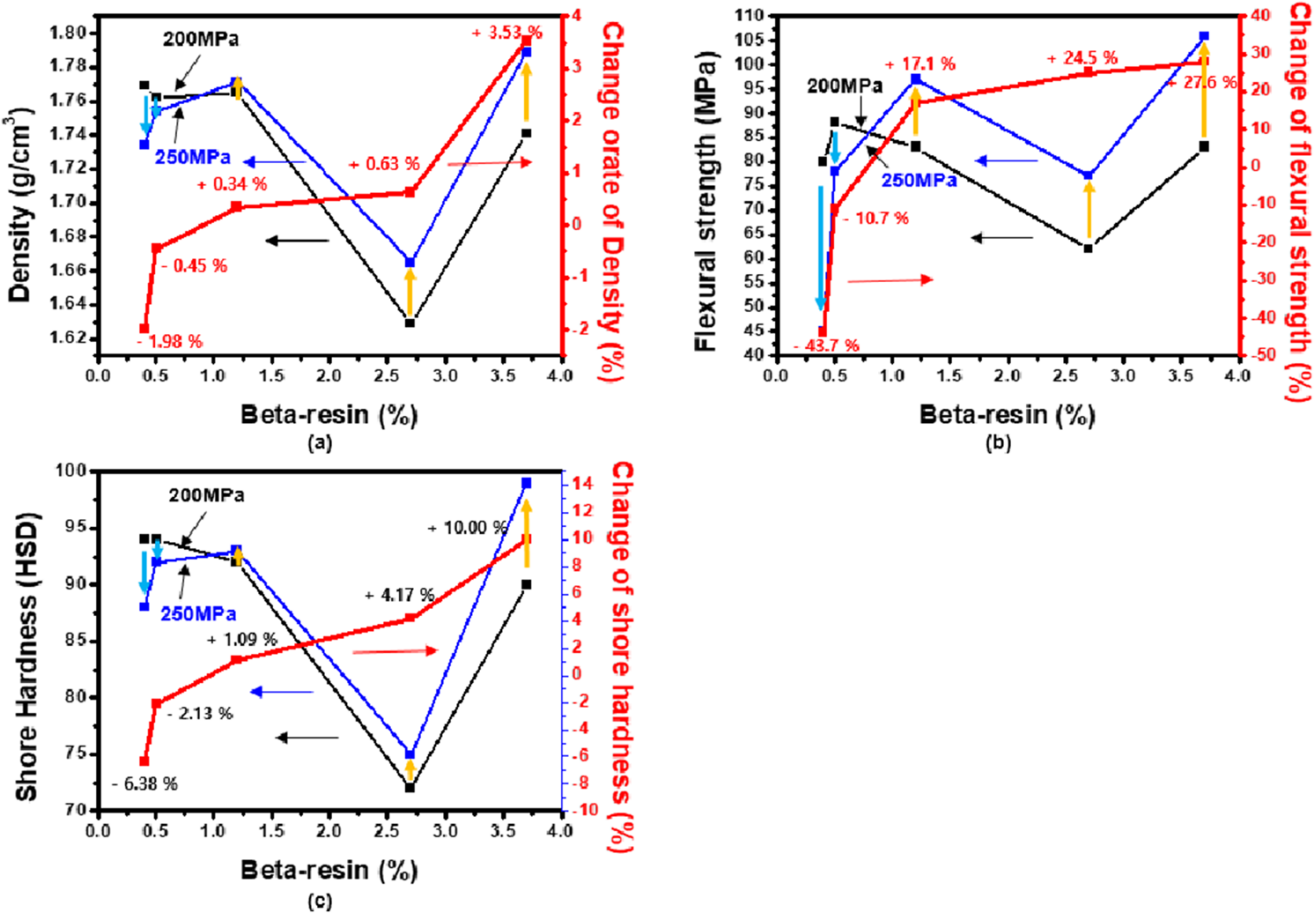
Changes in (**a**) density, (**b**) flexural strength, and (**c**) shore hardness of high-density carbon blocks after carbonization due to β-resin content.

**Figure 9:**
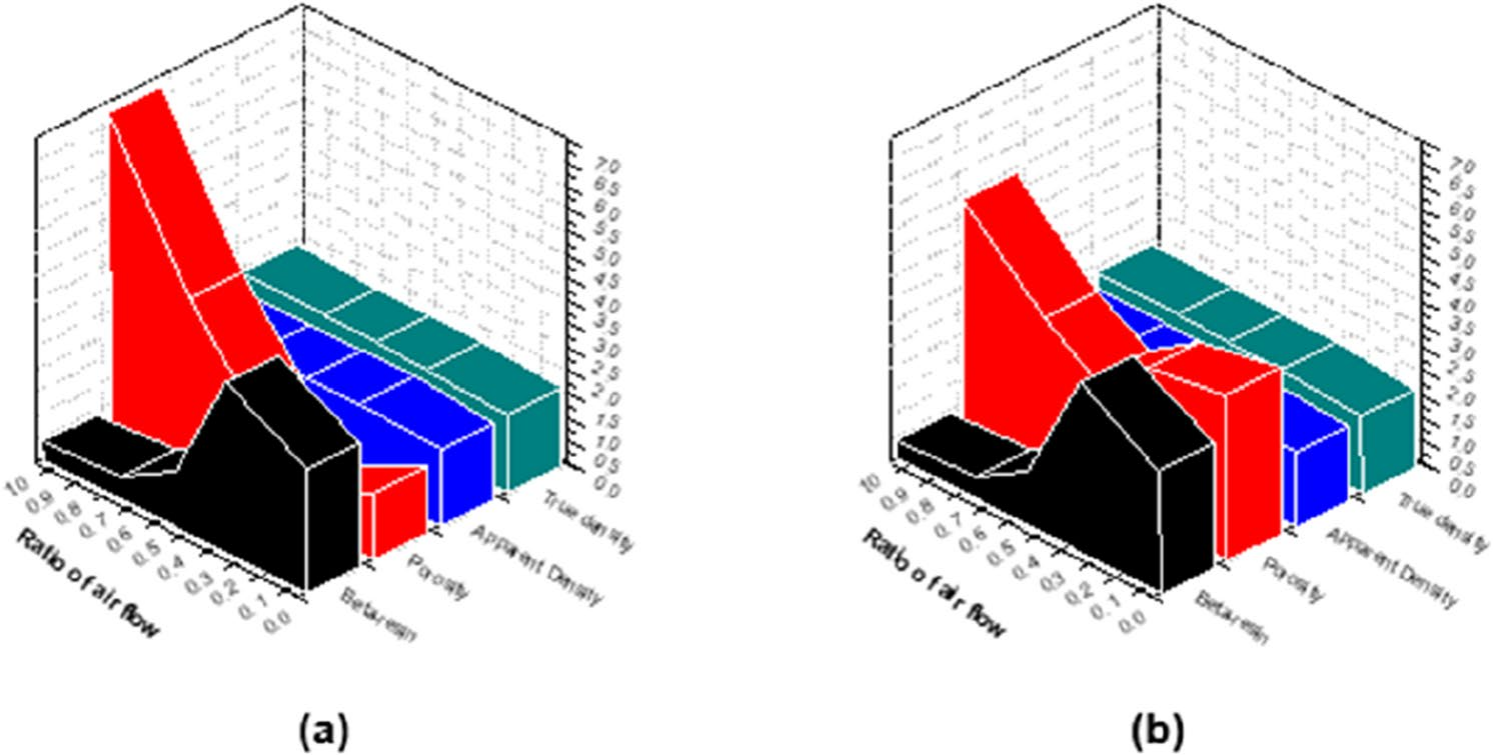
Change of porosity according to β-resin content when molding at (**a**) 200 MPa and (**b**) 250 MPa.

Figure 8 is a graph showing the mechanical properties of region II of Fig. 6 according to the β-resin content. As shown in Fig. 8a, in the case of density, the lower the β-resin content, the greater the decrease in density when molded at 250 MPa, and in the case of CB-N0A10, the decrease is 1.98%. On the other hand, in the case of CB-N10A0 and CB-N7A3, it increased by 2.45% and 3.53%, respectively. This is because the β-resin content is sufficient, and β-resin does not escape to the outside even when molded under high pressure and is also present inside to increase the density. In particular, CB-N7A3–250 showed the highest density at 1.79 g/cm^3^. As shown in Fig. 8b, the lower the β-resin content was, the greater the decrease in flexural strength, which was 44% in the case of CB-N0A10. On the other hand, in the case of CB-N10A0 and CB-N7A3, they increased by 25% and 28%, respectively, and in particular, CB-N7A3–250 had the highest flexural strength of 106 MPaAs, as shown in Fig. 8c. The lower the β-resin content was, the greater the decrease in shore hardness, which was 6.38% in the case of CB-N0A10. On the other hand, CB-N10A0 and CB-N7A3 increased by 4.17% and 10.00%, respectively, and CB-N7A3–250 showed the highest 99HSD shore hardness. Figure 9a and b show changes in porosity depending on the β- resin content of the high-density carbon blocks molded at 200 MPa and 250 MPa, respectively. CB-N0A10, which has the lowest β-resin content of 0.4 wt%, increased its porosity from 4.9% to 6.7%. On the other hand, the porosity of CB-N7A3, which had the highest β-resin content of 3.7 wt%, decreased from 3.3% to 0.6%. From this, it was found that the β-resin content has a great influence on the porosity of the carbon block, and this porosity has a great influence on the mechanical properties. The above-mentioned changes in mechanical properties can also be explained by this change in porosity.

These trends in mechanical properties can be explained through the relationship between β-resin and O/C shown in Fig. 3. At relatively low pressures below 200 MPa, higher mechanical properties are exhibited when the O/C ratio is high, whereas at high pressures above 200 MPa, the β-resin content plays an important role. Therefore, even if the O/C is low, it can be judged that the high-density carbon block with a high β-resin content exhibits higher mechanical properties. As expected in Fig. 3, when the β-resin content is low, the amount that protrudes to the outside when molding at a low pressure of under 200 MPa is small, and it can play a sufficient role in the O-functional group. However, when molding at a high pressure over 200 MPa, β-resin, which acts as a binder, protrudes to the outside, make higher porosity and the mechanical properties deteriorate. On the other hand, when the β-resin content is high, β-resin is sufficiently present inside the carbon particles even at a high pressure exceeding 200 MPa and make lower porosity. As a result, β-resin content determine the porosity of high-density carbon blocks and that affects change of mechanical properties at high molding pressure.

**Figure 10:**
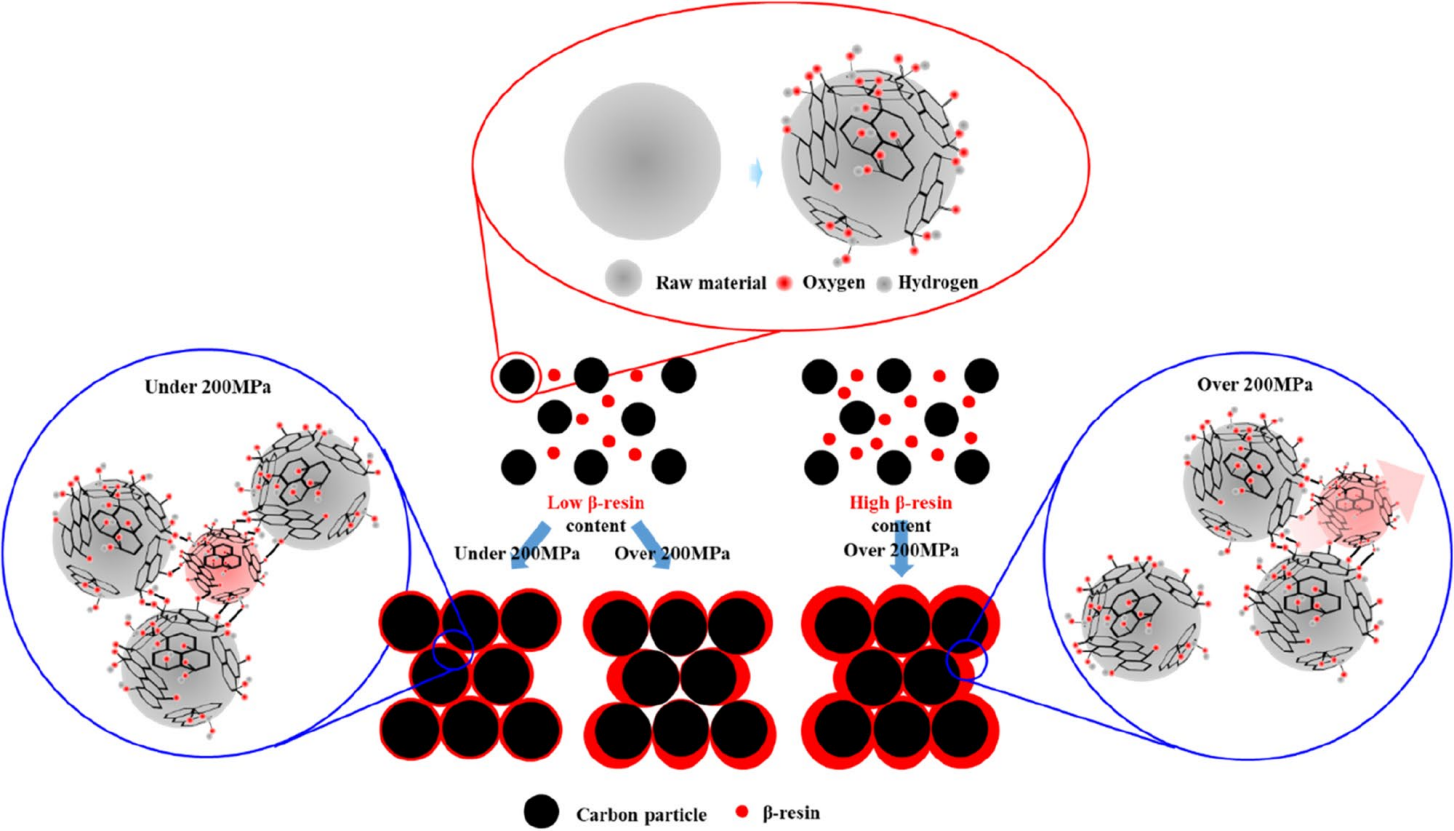
Effect of β-resin content on mechanical properties of high-density carbon blocks depending on molding pressure.

Based on the above results, when the molding pressure is varies based on 200 MPa, the schematic diagram of the mechanical properties of the high-density carbon block with β-resin content can be represented as in Fig. 10. When the molding pressure is under 200 MPa, carbon particles are bonded by acting as a sufficient binder without outflow of β-resin, and the mechanical properties increase as the molding pressure increases.

## Conclusion

In this study, carbon blocks were produced using binderless coke produced in a previous study, and the effects of the O/C ratio and β-resin content due to molding pressure on the mechanical properties were analyzed.

By up-taking oxygen into the binderless coke, the cross-linking reaction of volatile matter was induced to increase the molecular weight. As a result, the content of volatile matter can be reduced, the occurrence of swelling after carbonization can be prevented. It was found that β-resin and O/C have a cross-sectional relationship with each other, which has a great influence on the mechanical properties. The tendency of mechanical properties is displayed in the same way, and singular points appear, especially when molding at a pressure exceeding 200 MPa. This illustrates the fact that the β-resin content has a greater effect than the O/C ratio when molding at high pressure. As a result, the density of CB-N7A3–250, which appeared to have the best physical characteristics, was 1.79 g/cm^3^, the flexural strength was 106 MPa, and the shore hardness was 99 HSD. This is because the β-resin content is the highest at 3.7 wt%, and the physical properties steadily increase even when molded at high pressure.

## References

[1] Higinbotham H (1934). Colloida graphite as an adjunct lubricant for automobile engines. Proc. Inst. Automob. Eng..

[2] Bevilacqua M, Babutskyi A, Chrysanthou A (2015). A review of the catalytic oxidation of carbon-carbon composite aircraft brakes. Carbon.

[3] Miyazaki K, Hagio T, Kobayashi K (1981). Graphite and boron carbide composites made by hot-pressing. J. Mater. Sci..

[4] Lee SM, Kang DS, Kim WS, Roh JS (2014). Fabrication of isotropic bulk graphite using artificial graphite scrap. Carbon Lett..

[5] Yang Y, Hupta MC, Dudley KL, Lawrence RW (2005). A comparative study of EMI shielding properties of carbon nanofiber and multi-walled carbon nanotube filled polymer composites. Nanosci. Nanotechnol..

[6] Li N, Huang Y, Du F, He X, Lin X, Gao H, Ma Y, Li F, Chen Y, Eklund PC (2006). Electromagnetic interference (EMI) shielding of single-walled carbon nanotube epoxy composites. Nano Lett..

[7] Zhao Y, Liu Z, Wang H, Shi J, Zhang J, Tao Z, Guo Q, Liu L (2013). Microstructure and thermal/mechanical properties of short carbon fiber-reinforced natural graphite flake composites with mesophase pitch as the binder. Carbon.

[8] Liu Z, Guo Q, Shi J, Zhai G, Liu L (2008). Graphite blocks with high thermal conductivity derived from natural graphite flake. Carbon.

[9] Cho JH, Im JS, Kim MI, Lee YS, Bai YC (2020). Preparation of petroleum-based binder pitch for manufacturing thermally conductive carbon molded body and comparison with commercial coal-based binder pitch. Carbon Lett..

[10] T.Q. Li, Z.J. Hu, J.S. Wang, Y.M. Guo, C.Y. Wang, Influence of β-resin content and sintering conditions on the performance of high-density and isotropic carbon bulks derived from super-fine mesophase powder, *57 th International Astronautical Congress* (2006), 10.2514/6.IAC-06-C2.4.11

[11] Wang YG, Korai Y, Mochida I (1999). Carbon disc of high density and strength prepared from synthetic pitch-derived mesocarbon microbeads. Carbon.

[12] Gao Y, Song H, Chen X (2003). Self-sinterability of mesocarbon microbeads (MCMB) for preparation of high-density isotropic carbon. J. Mater. Sci..

[13] Zhou C, McGinn PJ (2006). The effect of oxygen on the processing of mesocarbon microbeads to high-density carbon. Carbon.

[14] Shen K, Huang ZH, Hu K, Shen W, Yu S, Yang J, Yang G, Kang F (2015). Advantages of natural microcrystalline graphite filler over petroleum coke in isotropic graphite preparation. Carbon.

[15] Weisshaus H, Kenig S, Siegmann A (1991). Effect of materials and processing on the mechanical properties of C/C composites. Carbon.

[16] Eslami Z, Yazdani F, Mirzapour MA (2015). Thermal and mechanical properties of phenolic-based composties reinforced by carbon fibres and multiwall carbon nanotubes. Composites: Part A.

[17] Im US, Kim JY, Lee BR, Peck DH, Jung DH (2019). Mechanical and electrical properties of MCMB/Chopped carbon fiber composite with different bead size. Sci. Rep..

[18] Planes E, Gloaguen F, Flandin L (2015). Optimizing formulations of polymer composite with high filler content: Application to bipolar plate. Compos. Sci. Technol..

[19] Zakaria MY, Sulong AB, Sahari J, Suherman H (2015). Effect of the addition of milled carbon fiber as a secondary filler on the electrical conductivity of graphite/epoxy composites for electrical conductive material. Compos. B.

[20] Mathur RB, Dhakate SR, Hupta DK, Dhami TL, Aggarwal RK (2008). Effect of different carbon fillers on the properties of graphite composite bipolar plate. J. Mater. Process. Technol..

[21] Fernández JJ, Figueiras A, Granda M, Bermejo J, Parra JB, Menéndez R (1995). Modification of coal-tar pitch by air-blowing II Influence on coke structure and properties. Carbon.

[22] Adamson MJ (1980). Thermal expansion and swelling of cured epoxy resin used in graphite/epoxy composite materials. J. Mater. Sci..

[23] Snead LL, Burchell TD, Katoh Y (2008). Swelling of nuclear graphite and high quality carbon fiber composite under very high irradiation temperature. J. Nucl. Mater..

[24] Xia X, Yih J, D’Souza NA, Hu Z (2003). Swelling and mechanical behavior of poly(N-isopropylacrylamide)/Na-montmorillonite layered silicates composite gels. Polymer.

[25] Mochida I, Shimizu K, Korai Y, Otsuka H, Sakai Y, Fujiyama S (1990). Preparation of mesophase pitch from aromatic hydrocarbons by the aid of HFBF3. Carbon.

[26] Zeng SM, Maeda T, Tokumitsu K, Mondori J, Mochida I (1993). Preparation of isotropic pitch precursors for general purpose carbon fibers (GPCF) by air blowing – II. Air blowing of coal tar, hydrogenated coal tar, and petroleum pitches. Carbon.

[27] Shen K, Huang ZH, Shen W, Yang J, Yang G, Yu S, Kang F (2015). Homogenous and highly isotropic graphite produced from mesocarbon microbeads. Carbon.

[28] Ragan S, Marsh HJ (1983). Use of oxidized needle-coke in the preparation of carbon artifacts. Mater. Sci..

[29] Lee SH, Lee SM, Im US, Kim SD, Yoon SH, Lee BR, Peck DH, Shul YG, Jung DH (2019). Preparation and characterization of high-spinnability isotropic pitch from 1-methylnaphthalene-extracted low-rank coal by co-carbonization with petroleum residue. Carbon.

[30] Park SJ, Lee SH, Lee SM, Park JW, Kim SS, Jung DH (2021). The effect of oxygen content in biinderless cokes for high-density carbon blocks from coal tar pitch. Materials.

[31] Fernandez JJ, Figueiras A, Granda M, Bermejo J, Menendez R (1995). Modification of coal-tar pitch by air-blowing-I. Variation of pitch composition and properties. Carbon.

[32] ASTM D2318–15, Standard Test Method for Quinoline-Insoluble (QI) Content of Tar and Pitch; ASTM International: West Conshohocken, PA, USA (2015), 10.1520/D2318-15

[33] ASTM D4072–98, Standard Test Method for Toluene-Insoluble (TI) Content of Tar and Pitch; ASTM International: West Conshohocken, PA, USA (2018), 10.1520/D4072-98R18

[34] Lee SM, Lee SH, Jung DH (2021). Surface oxidation of petroleum pitch to improve mesopore ratio and specific surface area of activated carbon. Sci. Rep..

[35] Fanjul F, Granda M, Santamaria R, Menendez R (2002). On the chemistry of the oxidative stabilization and carbonization of carbonaceous meshphase. Fuel.

[36] ASTM D790–17, Standard Test Methods for Flexural Properties of Unreinforced and Reinforced Plastics and Electrical Insulating Materials; ASTM International: West Conshohocken, PA, USA (2017), 10.1520/D0790-17

[37] ASTM D2240–15, Standard Test Method for Rubber Property—Durometer Hardness; ASTM International: West Conshohocken, PA, USA (2015), 10.1520/D2240-15R21

[38] Fitzer E, Huttner W, Manocha LM (1980). Influence of process parameters on the mechanical properties of carbon/carbon composites with pitch as matrix precursor. Carbon.

[39] Ming Z, Wen ZW, Dong ZH (2007). Effect of pressure on microstructures and mechanical properties of Al-Cu-based alloy prepared by squeeze casting. Trans. Nonferrous Met. Soc. China.

[40] Hu HL, Ko TH, Kuo WS, Chen ST (2006). Influence of adding mesocarbon microbeads into C.C Coomposites on microstructure and properties during carbonization. J. Appl. Polym. Sci..

[41] Norfolk C, Mukasyan A, Hayes D, McGinn P, Varma A (2004). Processing of mesocarbon microbeads to high-performance materials: Part I. Studies towards the sintering mechanism. Carbon.

